# Impact of the Benazir Nashonuma Programme on maternal and child health and nutrition in Pakistan: protocol for a prospective cohort study

**DOI:** 10.1136/bmjpo-2026-005047

**Published:** 2026-08-28

**Authors:** Shah Muhammad, Muhammad Muzzamil, Asma Abdul Malik, Imran Ahmed, Arjumand Rizvi, Muhammad Sajid, Muhammad Usman Ali, Ikram Maznani, Sajid B Soofi, Zulfiqar A Bhutta

**Affiliations:** 1 CoE in Women and Child Health, The Aga Khan University, Karachi, Sindh, Pakistan; 2 Paediatrics and Child Health, Aga Khan University, Karachi, Sindh, Pakistan; 3 Neonatology, Centre for Global Child Health, The Hospital for Sick Children, Toronto, Ontario, Canada

**Keywords:** Severe Acute Malnutrition, Child Health, Mortality, Infant, Low Birth Weight, Epidemiology

## Abstract

**Introduction:**

Maternal and child malnutrition continues to pose a considerable public health challenge in Pakistan, leading to increased instances of stunting, low birth weight and micronutrient deficiencies. The Government of Pakistan has instituted the Benazir Nashonuma Programme (BNP), a conditional cash transfer and specialised nutrition effort designed to assist pregnant and lactating mothers as well as young children. This prospective cohort study seeks to assess the effectiveness of the BNP in enhancing maternal and infant health and nutritional outcomes in the high-burden provinces of Sindh and Punjab.

**Methods and analysis:**

This longitudinal prospective cohort study will recruit 5446 pregnant women from BNP intervention and control regions and monitor mother-child pairs during pregnancy until 12 months post partum. The principal outcomes are the incidence of low birth weight and stunting in children at 6 and 12 months of age. Secondary outcomes encompass the prevalence of wasting; maternal nutrient intake; utilisation of reproductive health services; anaemia and iron deficiency anaemia in mothers and infants; infant and young child feeding practices; neurodevelopmental outcomes evaluated through the Bayley Scales of Infant and Toddler Development, 4th Edition; and programme-specific indicators such as the receipt and consumption of specialised nutritional food (Maamta and Wawamum). Dietary information will be gathered by 24-hour recalls while anthropometric measures and biospecimen analyses will be performed at regular intervals. Data will be examined using multivariable regression models while adjusting for confounding variables. The findings will provide evidence regarding the effectiveness of food-based and cash transfer interventions in enhancing health outcomes in resource-limited environments.

**Ethics and dissemination:**

Ethical approval has been obtained from the Aga Khan University Ethics Review and the Pakistan National Bioethics Committee. The cohort study and the associated quasi-experimental evaluation are registered at ClinicalTrials.gov. Written informed consent will be obtained from all participants. Study findings will be disseminated through peer-reviewed publications, policy briefs, stakeholder engagement and conference presentations.

**Trial registration number:**

NCT05836961, NCT06025786.

**WHAT IS ALREADY KNOWN ON THIS TOPIC**

**WHAT THIS STUDY HOPES TO ADD**

- This study will produce the first robust prospective cohort evidence on the Benazir Nashonuma Programme (BNP) effectiveness when implemented at scale through existing platforms.
- It will assess nutritional, anthropometric, biochemical and neurodevelopmental outcomes among mother–child pairs.
- It will bridge a significant evidence gap concerning cash transfer and nutrition support interventions in Pakistan.

**HOW THIS STUDY MIGHT AFFECT RESEARCH, PRACTICE OR POLICY**

- The findings will guide evidence-based decisions concerning the scale-up, enhancement and targeting of BNP and other cash transfer and nutrition support programmes, both in Pakistan and in similar low-resource contexts.

## Introduction

 Low birth weight (LBW), defined as a birth weight of less than 2500 g, is a critical public health challenge. It affects an estimated 20 million newborns globally each year, approximately 16% of all births, while 96.5% of these cases occur in low- and middle-income countries (LMICs).^1–3^ LBW contributes to 60–80% of neonatal deaths and is associated with increased morbidity, impaired neurodevelopment and heightened risk of chronic diseases in later life.^4 5^

Maternal nutrition throughout gestation is a critical determinant of birth outcomes. Pregnant women face a heightened risk of multiple micronutrient deficiencies, especially in developing countries.^6^ The nutritional status of a woman before and during pregnancy profoundly influences fetal development. Maternal nutrition influences placental function and food supply to the fetus, with insufficient nutrition often resulting in intrauterine growth restriction, premature birth and other adverse maternal and neonatal consequences. Maternal malnutrition is a critical concern in LMICs, as numerous women in these regions experience food shortages, compounded by cultural beliefs and insufficient healthcare.^7–9^

Deficiencies in macronutrients and micronutrients are recognised to have detrimental impacts; macronutrients supply energy for fetal metabolism. Energy shortage resulting from inadequate calorie intake might result in restricted fetal growth. Additional minerals that play crucial roles in embryonic development encompass iron, folic acid, zinc and iodine.^7^ For instance, pregnant women with iron deficiency anaemia (IDA) experience reduced oxygen transport to the fetus, resulting in an increased likelihood of LBW.^10^

The WHO reports the prevalence of anaemia among pregnant women. In 2023, 35.5% of pregnant women aged 15–49 years experienced anaemia globally, categorising it as a significant public health issue within this demographic.^11^ The predominant kind of anaemia in pregnant women is IDA. The recommended daily iron consumption for a typical adult female is approximately 14.8 mg, increasing to about 30 mg/day during pregnancy. This is because iron is essential not only for normal biological activities but also for enhancing red blood cell synthesis and expanding plasma volume, thereby sustaining the fetal-placental circulation.^12^ The National Nutrition Survey (NNS) of Pakistan reports a high burden of iron deficiency among pregnant women in rural areas, affecting 44.3% of this population.^13^

Approximately 16% of live births worldwide are affected by LBW, with a higher prevalence in LMICs (19%) compared with high-income settings (7%). In Pakistan, LBW prevalence ranges from about 19% in urban areas to as high as 32% in rural populations, contributing substantially to under-five mortality, childhood stunting and long-term health inequities.^14^ LBW significantly increases the chance of neonatal mortality and, for those who survive, sets a life trajectory characterised by stunted growth, diminished cognitive development and heightened susceptibility to chronic diseases in adulthood, including obesity and diabetes.^4^

The effects of LBW on personal growth and cognitive development also have significant implications for national productivity and economic growth, correlating with lower educational attainment and reduced income potential.^15^ The economic consequences are significant, with malnutrition projected to cost Pakistan over US$7.6 billion per year, representing 3% of its gross domestic product.^16^

There is a unique chance to improve maternal and child nutrition and health outcomes during the first 1000 days of a child’s life, which begins at conception and ends at 2 years of age.^17^ Supplementing pregnant women in undernourished populations with balanced energy-protein (BEP) can lower the risk of stillbirth and the incidence of small-for-gestational-age (SGA) babies, according to the WHO’s antenatal care (ANC) guidelines.^18 19^

An increase in human development outcomes can be achieved by conditional cash transfers (CCT) that are tied to the utilisation of maternal and child health services.^20 21^ Evaluations and trials of programmes in Pakistan have shown a decrease in stunting and wasting in children, which is consistent with evidence that lipid-based nutritional supplements improve mother and child health and nutrition.^22 23^

## Intervention: Benazir Nashonuma Programme

**Figure 1:**
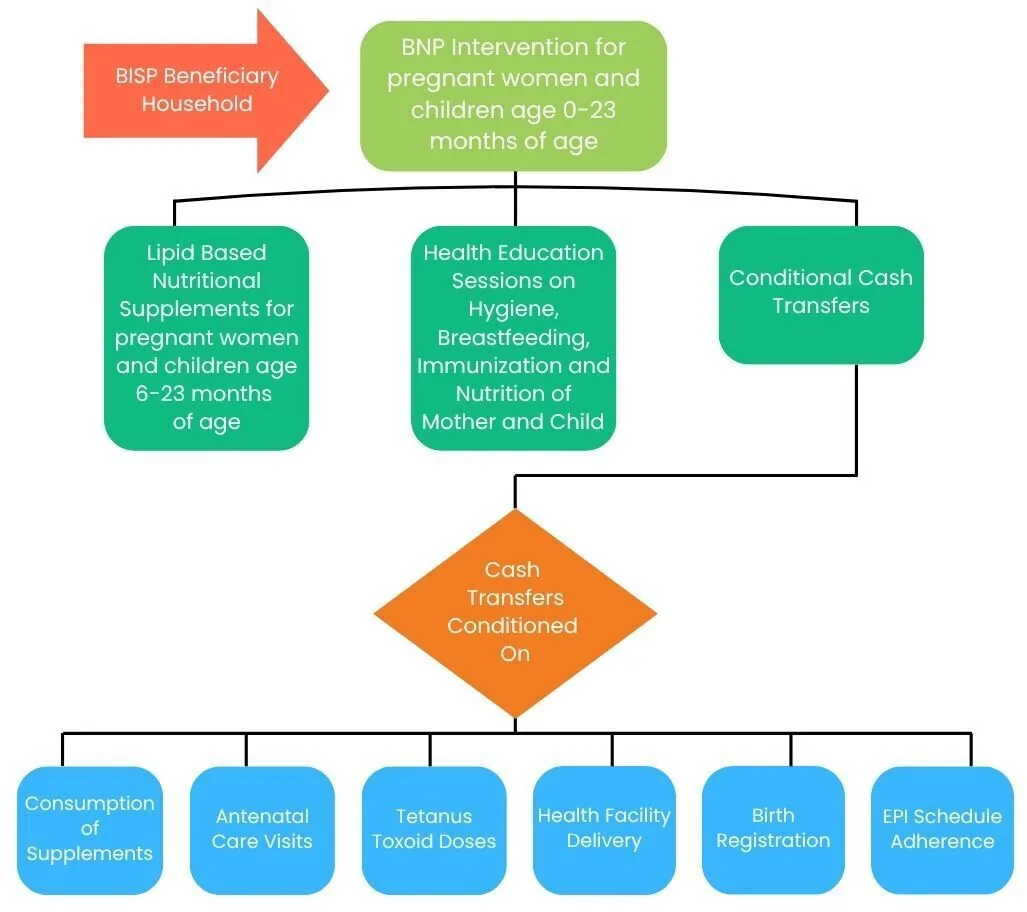
Flow of intervention components for BNP beneficiaries. BISP, Benazir Income Support Programme; BNP, Benazir Nashonuma Programme.

In order to reduce Pakistan’s high burden of stunting, the Benazir Income Support Programme (BISP) launched the Benazir Nashonuma Programme (BNP) in conjunction with the World Food Programme in August 2020. As the country’s largest social safety net, BISP offers quarterly cash transfers to disadvantaged women identified by the National Socio-Economic Registry, a Proxy Means Test (PMT)-based system targeting the poorest 20% of households.^24 25^ The BNP is a conditional health and nutrition intervention for pregnant and breastfeeding women and children aged 6–24 months enrolled in BISP,^24^ administered through facilitation centres integrated within government health institutions across 157 districts, including mobile centres in Sindh.^26^ Beneficiaries receive lipid-based nutritional supplements alongside CCTs linked to supplement use, ANC, tetanus vaccination and participation in health and nutrition awareness sessions, with compliance verified at facilitation centres prior to biometric cash disbursement.^27^ Figure 1 represents the BNP flow.

## Study aims and objectives

This study aims to evaluate the impact of an integrated intervention comprising BEP supplementation and CCTs, alongside targeted antenatal services and behaviour change communication, on maternal and newborn health outcomes. Specifically, the study will assess the effectiveness of this package in reducing the incidence of LBW, stunting and other adverse outcomes including SGA, preterm birth and pregnancy-related complications. Specific study objectives include:

### Primary objectives

- To assess the impact of the BEP supplementation and CCTs/BNP nutrition intervention on the incidence of babies born with LBW in low-resource setting of Sindh and Punjab, Pakistan.
- To determine the incidence of stunting among infants at 6 and 12 months of age in low-resource setting of Sindh and Punjab, Pakistan.

### Secondary objectives

- To assess the impact of BEP supplementation and CCTs/BNP nutrition intervention on risk of wasting at 6 and 12 months of age.
- To assess the impact of BEP supplementation and CCTs/BNP nutrition intervention on neurodevelopmental outcomes.
- To assess the impact of BEP supplementation and CCTs/BNP nutrition intervention on gestational weight gain.
- To conduct a cost-effectiveness analysis of the BNP.

## Methodology

### Study design

This is a prospective cohort study. The study will compare maternal and child health outcomes between two cohorts of pregnant women: one enrolled in the BNP and another not enrolled in the programme. Both cohorts will be drawn from the same geographic areas to ensure contextual comparability.

### Study setting

The study will be conducted in two districts of Pakistan: Rajanpur (Punjab) and Dadu (Sindh). These districts were selected based on the operational status of BNP centres in each tehsil. A BNP centre was considered functional if it was operational at a Tehsil Headquarters facility with active registration system, availability of specialised nutritious foods (Maamta/Wawamum), a dedicated SBCC corner and assigned facility in-charge. Districts were selected where all tehsils had at least one such centre to ensure consistent programme delivery. The exposed cohort will be drawn from women attending functional BNP centres, while the unexposed cohort will be identified among BISP beneficiaries in the same districts who are not enrolled in BNP.

### Study timelines

March 2023–June 2026.

### Study population and eligibility criteria

The study population will be pregnant women of reproductive age (15–49 years) who are registered beneficiaries of the BISP. The exposed group will include pregnant women enrolled in BNP, while the unexposed group will comprise pregnant BISP beneficiaries who are not enrolled in the BNP at the time of recruitment. Both groups will be drawn from the same geographic areas to ensure comparability.

Eligible participants must meet the following inclusion criteria:

1. Pregnant women aged 15–49 years.
2. In their first or second trimester.
3. Registered as BISP beneficiaries.

Participants will be excluded if they have plans to migrate or relocate from the study area for a period exceeding 3 months during the study period. Furthermore, pregnant women in the unexposed group who are already consuming specialised nutritious food from sources other than BNP at the time of enrolment will also be excluded.

### Study duration

Participant recruitment will be completed within 12 months of study initiation. The total follow-up duration will span 3 years, from pregnancy identification to 12 months post-delivery. This is a closed cohort study. Attrition is anticipated to be higher among the unexposed group, as some women may enrol in BNP during the study period, altering their exposure status.

### Intervention

BNP facilitation centres, established within public health facilities at the subdistrict level in intervention clusters, serve as the focal points for delivering programme interventions. Beneficiaries receive specialised nutritious food and CCTs, contingent on supplement consumption, ANC attendance, tetanus vaccination and participation in health and nutrition education sessions.

### Specialised nutritious food

The specialised nutritious food includes locally produced lipid-based nutrient supplements for both pregnant and lactating women (Maamta) and children aged 6–23 months (Wawamum). These ready-to-use products do not require refrigeration and have a long shelf life.

- *Maamta* (75 g/day): a nutrient-dense lipid-based supplement designed for pregnant and lactating women, with over 50% of its energy derived from lipids and less than 25% from protein. It is formulated with chickpeas, vegetable oils, dry skimmed milk, sugar, vitamins and minerals and has been shown to improve maternal nutritional status in undernourished populations (table 1).
- *Wawamum* (50 g/day): a medium-quantity lipid-based supplement for children aged 6–23 months, providing approximately 255–260 kcal/day, covering most micronutrient needs and about one-quarter of the daily energy requirement for this age group (table 2).

**Table 1:** Composition of 75 g LNS-PLW supplement (Maamta)

Nutrient	Amount	Nutrient	Amount
Energy	400 kcal	Vitamin E	12 mg
Protein	10.5 g	Vitamin K	20.2 μg
Lipids	24 g	Vitamin C	45 mg
Sodium	<203 mg	Vitamin B_1_ (thiamine)	0.75 mg
Potassium	675 mg	Vitamin B^2^ (riboflavin)	1.57 mg
Calcium	400 mg	Vitamin B_3_ (niacin)	9.75 mg
Phosphorus	337 mg	Vitamin B_5_ (pantothenic acid)	3 mg
Magnesium	112 mg	Vitamin B_6_	1.35 mg
Iron	7.5 mg	Vitamin B_7_ (biotin)	45 μg
Zinc	8.2 mg	Vitamin B_9_ (folates)	247 μg
Copper	1.0 mg	Vitamin B_12_	2.0 μg
Selenium	15 μg	Dry skimmed milk protein	2.7 g
Iodine	75 μg	Fatty acid n-6	1.95 g
Manganese	0.9 mg	Fatty acid n-3	0.22 g
Vitamin A	412 μg	Fibre content	<5%
Vitamin D	11.2 μg	Moisture	<2.50%

**Table 2:** Composition of 50 g LNS-MQ supplement (Wawamum)

Nutrient	Amount	Nutrient	Amount
Energy	255 kcal	Vitamin E	8 mg
Protein	5.5 g	Vitamin K	13.5 μg
Lipids	13 g	Vitamin C	30 mg
Sodium	<135 mg	Vitamin B_1_ (thiamine)	0.5 mg
Potassium	450 mg	Vitamin B_2_ (riboflavin)	1.05 mg
Calcium	267 mg	Vitamin B_3_ (niacin)	6.5 mg
Phosphorus	225 mg	Vitamin B_5_ (pantothenic acid)	2.0 mg
Magnesium	75 mg	Vitamin B_6_	0.9 mg
Iron	5 mg	Vitamin B_7_ (biotin)	30 μg
Zinc	5.5 mg	Vitamin B_9_ (folates)	165 μg
Copper	0.7 mg	Vitamin B_12_	1.35 μg
Selenium	10 μg	Dry skimmed milk protein	1.8 g
Iodine	50 μg	Fatty acid n-6	1.3 g
Manganese	0.6 mg	Fatty acid n-3	0.15 g
Vitamin A	275 μg	Fibre content	5.0%
Vitamin D	7.5 μg	Moisture	<2.50%

### Cash transfers

Cash support is provided quarterly:

- *PKR2500 *(*~US$8.86*) for pregnant women until childbirth.
- Post-delivery: *PKR3000* (*~US$10.63*) for a girl child and *PKR2500* for a boy child.

### Eligibility and conditionalities

To qualify for cash benefits, participants must:

- Consume at least 90% of their specialised nutritious food allocation, verified by returning empty sachets.
- Attend three ANC visits.
- Receive two doses of tetanus toxoid vaccine.
- Participate in 11 health and nutrition education sessions (3 during pregnancy, 8 postpartum) covering topics such as hygiene, breastfeeding and dietary diversity.
- Deliver in a health facility.
- Ensure birth registration and adherence to the national immunisation schedule (EPI).

### Participant enrolment

Pregnant women in their first or second trimester will be identified for study enrolment through two primary sources. For the exposed group, participants will be identified from the registration records at BNP facilitation centres. These women will then be approached at the household level within their communities for enrolment. For the unexposed group, eligible women will be selected from the BISP beneficiary lists, with verification conducted through household visits by our study teams.

Written informed consent will be obtained from all eligible participants prior to enrolment. Gestational age will be determined using the date of the last menstrual period (LMP) and verified through ultrasound reports where available. For participants without a prior ultrasound, referrals will be made to the nearest government health facility to facilitate this examination.

All enrolled women will be monitored monthly throughout their pregnancy and at the time of delivery. Additionally, postnatal follow-up for the mother-child dyad will continue monthly until the child attains 12 months of age. Subsequent activities will be carried out by an autonomous data collection team to guarantee objectivity and the integrity of the data. To address attrition, women intending to relocate from the study area for a duration exceeding 3 months will be excluded during the enrolment process. In cases of dropout, the reason for discontinuation whether due to migration, voluntary withdrawal or crossover to BNP enrolment will be thoroughly documented and considered during the data analysis and interpretation of results.

### Data collection and follow-up time points

Women will be enrolled at their households in the communities. Their baseline data will be collected on sociodemographic characteristics, dietary intake (using the 24-hour multiple-pass recall method), maternal anthropometry (weight, height and mid-upper arm circumference), women empowerment and baseline biochemical assessments for IDA. Gestational age will be determined based on the LMP and verified using ultrasound when available. If not already done, participants will be referred to the nearest government health facility for an ultrasound.

Validated survey tools adapted from the Pakistan Demographic and Health Survey, NNS 2018, Food Insecurity Experience Scale (FIES), Infant and Young Child Feeding (IYCF) questionnaire, Minimum Dietary Diversity for Women (MDDW) and the national immunisation schedule will be used. All tools will be translated into Urdu and back-translated to ensure accuracy. A mobile application developed in Java with an SQLite backend will facilitate computer-assisted personal interviewing. Data will be securely transmitted to the Aga Khan University (AKU) data centre and stored using Microsoft SQL Server 2012. Pilot testing of questionnaires and the data collection application will precede full-scale data collection.

Pregnant participants will be followed monthly to monitor maternal weight, MUAC, dietary intake, utilisation of reproductive health services and morbidity status. In the exposed cohort, compliance with specialised nutritious food supplementation will also be assessed through self-reported questionnaire. Haemoglobin levels will be reassessed in the third trimester to monitor IDA.

### Delivery and birth assessment

Birth outcomes will be captured within 48 hours of birth through delivery notification system. This system will involve incentives for families and community health workers who inform about the birth timely. This will be complemented with daily reminder calls by the study teams near the expected date of delivery. Newborns will be assessed for birth weight, birth length, MUAC and fronto-occipital circumference. Spot haemoglobin of the mother will be measured using the HemoCue 301+ device.

### Postnatal follow-ups

Mothers will continue to be followed monthly for 6 months for anthropometry, specialised nutritious food compliance (in the exposed group) and morbidity surveillance. Infants will be followed monthly for anthropometric measurements (weight, length, MUAC and fronto-occipital circumference), IYCF indicators, immunisation status, morbidity, mortality, and from 6 months onward, specialised nutritious food compliance. Infant biochemical investigations (haemoglobin, ferritin, C-reactive protein (CRP) and alpha-1-acid glycoprotein (AGP)) and neurodevelopmental outcomes will be assessed at 12 months of age.

### Anthropometric measurements

Anthropometry will follow Food and Nutrition Technical Assistance III and SMART guidelines. Women’s weight will be measured using Seca 874 U electronic scales, and children’s weights with infant scales to the nearest 0.1 kg. Length will be measured using appropriate boards to the nearest 0.1 cm. MUAC tapes standard for women and colour coded for children will be used. All measurements will be taken twice; discrepancies beyond set thresholds will prompt a third measurement, with the mean of the two closest values used as the final reading. Add fronto-occipital measurements, too.

### Biochemical assessment

Blood samples will be collected from mothers at enrolment and during the third trimester follow-up, and from infants at 6 and 12 months of age. Standard venipuncture procedures with proper personal protective equipment will be followed. Haemoglobin will be measured immediately using the HemoCue 301+ system. For ferritin, CRP and AGP, 1.5 mL of serum will be collected using gel top vacutainers and analysed using the COBAS C311 analyser.

### Team structure and training

16 data collection teams per district will be deployed, each consisting of two female data collectors. Centralised training will cover questionnaire administration, anthropometry per SMART methodology, electronic data collection and laboratory protocols. Phlebotomists will receive targeted training in blood collection and biosafety.

### Data management and security

Data will be uploaded daily to the AKU data centre. In areas lacking internet connectivity, data will be backed up manually by team leaders. Role-based access control will govern entry to the AKU repository, and daily backups will be stored in a remote secure location.

### Quality assurance

Field supervisors, a study manager and an independent verification team will oversee data quality. Regular refresher trainings will be held monthly, then bimonthly. Random household revisits will be conducted to validate data accuracy. Anthropometric data will be checked using ENA-SMART software for plausibility.

### Neurodevelopmental assessment

A subsample of children will undergo neurodevelopmental evaluation using the Bayley Scales of Infant and Toddler Development, 4th Edition (BSID-IV), at 12 and 24 months. This tool assesses cognitive, motor, language, social-emotional and adaptive behaviour domains, providing comprehensive insights into child development.

### Study outcomes

- *Maternal outcomes*
  - Maternal weight gain and body mass index during pregnancy.
  - Maternal dietary diversity and mean nutrient intake (energy in kcal).
  - Utilisation of reproductive health services.
  - Incidence of pregnancy-related morbidities.
  - Prevalence of IDA.
  - Women’s empowerment during pregnancy and lactation.
- *Infant outcomes*
  - Prevalence of wasting among infants at 6 and 12 months.
  - IYCF practices.
  - Neurodevelopmental milestones using BSID-IV at 12 and 24 months.
  - Incidence of anaemia and morbidity.
  - Age-appropriate immunisation coverage.
- *Programme utilisation and household outcomes*
  - Uptake and utilisation of specialised nutritious foods (Maamta/Wawamum) by mothers and children.
  - Household food insecurity measured using the FIES.

### Sample size

A minimum of 5446 pregnant women, 2223 in the exposed group and 3223 in the unexposed group, will be enrolled to detect a 20% reduction in LBW (baseline prevalence 20.1%) and a 15% reduction in stunting at 12 months of age (baseline prevalence 36.7%) (table 3). This sample size is calculated to achieve 80% power at a 5% level of significance, accounting for an anticipated 30% attrition rate.

**Table 3:** Estimated sample sizes for detecting key maternal and child health outcomes between BNP exposed and unexposed cohorts

Outcomes	Baseline prevalence/mean (SD)	Target reduction/change	Power (%)	Total sample	Exposed	Unexposed
Low birth weight	20.1%	20%	80	5446	2223	3223
Stunting at 12 months	36.7%	15%	80	4474	1737	2737
NDA—cognition	92±14	+5 points	80	648	274	374
NDA—language	70.5±13.5	+5 points	80	968	434	534
NDA—motor	79±13	+5 points	80	742	321	421

BNP, Benazir Nashonuma Programme; NDA, neurodevelopmental assessment.

The unexposed cohort has been deliberately oversampled by 1000 participants to account for potential exposure crossover, that is, unexposed women enrolling in the Nashonuma programme during the study period, which could result in higher attrition or contamination in this group. Additionally, a subsample of 586 children will undergo neurodevelopmental assessments at 12 and 24 months of age.

### Plan of analysis

Descriptive statistics will be used to compare baseline characteristics between the exposed and unexposed cohorts. Continuous variables will be presented as means±SDs or medians with IQRs, as appropriate. Categorical variables will be summarised as frequencies and percentages.

Continuous outcomes will be analysed using linear regression, while binary outcomes, including LBW and stunting at 6 and 12 months of age, will be analysed using logistic regression.

To account for baseline differences between groups, propensity scores will be calculated based on predictors of BNP enrolment. These scores will be used to create matched cohorts, thereby improving comparability between the exposed and unexposed groups.

To assess longitudinal outcomes such as child growth, dietary intake and maternal weight gain, linear mixed models will be employed. These models will incorporate time-varying effects, individual-level random effects and interaction terms to examine how the impact of BNP exposure evolves over time. Timing of exposure will be included as a confounding variable in all relevant models. All analyses will adjust for potential confounders. Interaction effects, if present, will be reported. Statistical analyses will be conducted using STATA V.17, and dietary data will be processed using Nutritionist Pro software V.7.5.

A cost-effectiveness analysis will be conducted alongside the cohort study using programme costs obtained from BISP administrative records and effectiveness estimates from this cohort to generate incremental cost-effectiveness ratios.

## Study outcomes

### Primary outcomes

The primary outcomes of the cohort study are:

- *Prevalence of LBW*: defined as a birth weight of less than 2500 g within 48 hours of delivery. This will be analysed as a continuous, dichotomous (LBW vs normal birth weight (NBW)) and ordinal variable (NBW ≥2500 g; LBW <2500 g; very LBW <1500 g; extremely LBW <1000 g).
- *Prevalence of stunting*: assessed at 6 and 12 months of age, defined as height-for-age z-score (HAZ) ≤−2 SD based on WHO growth standards.

### Secondary outcomes

- *Maternal outcomes*
  - *Mean nutrient intake* (*kcal*): assessed via 24-hour dietary recall at enrolment and quarterly until 6 months post partum.
  - *MDDW*: proportion of women of reproductive age consuming ≥5 of 10 food groups in the previous 24 hours (FAO^28^).
  - *Uptake of reproductive health services*, including:
    1. At least eight ANC visits.
    2. At least one of each during pregnancy: ultrasound, blood pressure check, weight check, urine and blood samples, family planning counselling.
    3. Receipt of iron-folic acid supplementation.
    4. Facility-based delivery attended by skilled personnel.
    5. Postnatal check within 2 days of delivery.
  - *IDA in pregnancy*: assessed at enrolment and during the third trimester. Diagnostic criteria: haemoglobin <110 g/L, serum ferritin ≤30 ng/mL, MCV <95 fL.
- *Infant outcomes*
- *Anthropometric indicators* (at 6 and 12 months):
  - *Stunting* (HAZ ≤−2 SD).
  - *Wasting* (weight-for-height z-score (WHZ) ≤−2 SD).
  - Mean HAZ and WHZ scores.
- *IDA*: assessed at 6 and 12 months. Defined as haemoglobin <110 g/L and serum ferritin <15 µg/L.
- *IYCF indicators (0–12 months)* (WHO and UNICEF^29^), including:
  1. Ever breastfed.
  2. Breastfed within 1 hour of birth.
  3. Exclusive breastfeeding (0–5 months).
  4. Bottle feeding.
  5. Introduction of solids (6–8 months).
  6. Minimum acceptable diet (6–23 months).
  7. ≥2 milk feeds for non-breastfed children (6–23 months).
  8. Minimum dietary diversity (five of eight food groups).
  9. Minimum meal frequency.
  10. Consumption of sweet beverages.
  11. Consumption of eggs/flesh foods.
  12. No fruit/vegetable consumption.
- *Neurodevelopment*: assessed at 12 and 24 months using the BSID-IV. Composite scores will be reported for cognitive, language, motor, social-emotional and adaptive behaviour domains.

### Programme-related outcomes

- *Supplementary nutrition food (SNF) coverage and compliance*
  - Proportion of pregnant and lactating women (up to 6 months post partum) who received and consumed Maamta in the past 4 weeks.
  - Proportion of children aged 6–12 months who received and consumed Wawamum in the past 4 weeks.
- *Health and nutrition service uptake*
  - Proportion of women receiving health education during their last visit to the facilitation centre.
  - Proportion of children whose anthropometry (weight, height, MUAC) was measured during their last visit.

### Other outcomes

- *Household food insecurity*: assessed using the FIES (FAO, 2021). Prevalence of food insecurity will be reported by severity (mild, moderate and severe) over the past 12 months.

## Ethical considerations

As the efficacy and effectiveness of the commodity are well established and the study aims to assess the impact of BNP on nutritional outcomes at the population level, ethical oversight is maintained through annual progress reviews with AKU Ethics Review Committee. The cohort study is registered on ClinicalTrials.gov under the identifier *NCT05836961* and the quasi-experimental component under *NCT06025786*.

Before enrolment, written informed consent will be obtained from all participants. The consent process will communicate the voluntary nature of participation, the study objectives, the procedures involved and the right to withdraw at any point without any repercussions. Confidentiality and data protection are central to this study. All data will be stored securely with password protection and restricted access limited to authorised study personnel only. Personal identifiers will be removed during data processing and analysis to ensure anonymity. Data will be handled in compliance with ethical standards throughout the research process, including publication and dissemination of findings.

## Conclusion

This prospective cohort study aims to generate rigorous evidence on the effectiveness of the BNP in addressing the persistent and complex burden of maternal and child malnutrition in Pakistan, with a particular focus on the reduction of LBW and childhood stunting. Recognising the critical importance of the first 1000 days of life, the study assesses a comprehensive range of maternal, newborn and infant health outcomes spanning dietary diversity, nutrient intake, reproductive health service uptake, anaemia, IYCF practices, neurodevelopment and programme-specific indicators such as compliance and utilisation of specialised nutritious foods.

Implemented in a real-world setting and building on promising global evidence from food-based interventions in low-income contexts, this study is uniquely positioned to evaluate the impact of BNP in a geographically and socioeconomically diverse population. It also accounts for important contextual factors, such as household food insecurity, health system access and behavioural patterns that shape nutritional outcomes.

The findings of this study will have broad relevance and potential generalisability for similar low- and middle-income settings. By rigorously evaluating both biological and behavioural outcomes through longitudinal follow-up, this study will contribute valuable insights into the long-term effectiveness of targeted nutritional and health interventions during pregnancy and early childhood. Importantly, the results will inform future programmatic decisions and policy directions for integrating and institutionalising BNP within Pakistan’s national health service delivery platforms, ensuring a sustained impact on maternal and child health outcomes. This research will help validate the effectiveness of BNP and strengthen the evidence base for designing and scaling comprehensive, equity-driven and nutrition-sensitive interventions to improve the health trajectories of women and children in underserved populations.

## Data Availability

No data are available.
