# Supplementary material for: Impact of the Benazir Nashonuma Programme on maternal and child health and nutrition in Pakistan: protocol for a prospective cohort study
Source: BMJ Paediatr Open. 2026 Aug 28;10(1):e005047. doi: 10.1136/bmjpo-2026-005047 (PMC13536015; doi:10.1136/bmjpo-2026-005047)
Supplement: Supplementary file 1 [file bmjpo-2026-005047.reviewer_comments.pdf]

PEER REVIEW HISTORY

BMJ Paediatrics Open publishes all reviews undertaken for accepted manuscripts. Reviewers are asked to complete a checklist review form and are provided with free text boxes to elaborate on their assessment. These free text comments are reproduced below.

ARTICLE DETAILS

**TITLE (PROVISIONAL)**	Impact of the Benazir Nashonuma Programme on Maternal and Child Health and Nutrition in Pakistan: Protocol for a Prospective Cohort Study
**AUTHORS**	Muhammad , Shah<br>Muzzamil, Muhammad<br>Abdul Malik, Asma<br>Ahmed, Imran<br>Rizvi, Arjumand<br>Sajid, Muhammad<br>Ali, Muhammad Usman<br>Maznani, Ikram<br>soofi, sajid<br>Bhutta, Zulfiqar

VERSION 1 – REVIEW

**REVIEWER NAME**	Patwari, Ashok
**REVIEWER AFFILIATION**	Lady Hardinge Medical College, Pediatrics
**REVIEWER CONFLICT OF INTEREST**	No Conflict of Interest
**AI DISCLOSURE**	No, I have not used AI
**DATE REVIEW RETURNED**	24-Jun-2026

**GENERAL COMMENTS**	It is a very good study protocol aimed to study a conditional health and nutrition intervention. However, I have the following queries that need to be clarified:<br><br>(1). Study Setting: It is stated that "The study will be conducted in two districts of Pakistan: Rajanpur (Punjab) and Dadu (Sindh).These districts were selected based on the operational status of BNP centers in each Tehsil."<br><br>The authors need to explain the criteria used for assessing operational status of BNP, and what is the definition of a functional BNP center.<br><br>(2). Criteria for cash transfers is different in the two cohorts. The protocol mentions:" BISP offers quarterly cash transfers to disadvantaged women identified by the National Socio-Economic Registry.."<br><br>Do the disadvantaged women in BSP group get two types of cash transfers?<br><br>How are we comparing the two cohorts when some of the study subjects in the control group enrolled in BISP are not eligible for cash transfers, while as everybody enrolled in BSP is, even though there are some specific conditions.

	(3).Is there any tool available to assess how the cash transfers are spent by the families because differences in the socio-economic status and other factors within the families can influence expenditure of this additional benefit.<br><br>(4). It appears the study is expected to be completed in June 2026. Was any Data Safety Monitoring Board (DSMP) meeting held during the study period ? If yes, was there any concern raised by the board ?

VERSION 1 – AUTHOR RESPONSE

Reviewer Comments Response Sheet:
Reviewer: 1
Dr. Ashok Patwari, Lady Hardinge Medical College

1. Study Setting: It is stated that "The study will be conducted in two districts of Pakistan: Rajanpur (Punjab) and Dadu (Sindh). These districts were selected based on the operational status of BNP centers in each Tehsil."
The authors need to explain the criteria used for assessing operational status of BNP, and what is the definition of a functional BNP center.

Response 1: Functional BNP Center
A BNP center was considered functional if it was operational at a Tehsil Headquarters facility with active registration system, availability of specialized nutritious foods Maamta/Wawamum), a dedicated SBCC corner, and assigned facility in-charge. Districts were selected where all Tehsils had at least one such center to ensure consistent program delivery.

2. Criteria for cash transfers is different in the two cohorts. The protocol mentions:" BISP offers quarterly cash transfers to disadvantaged women identified by the National Socio-Economic Registry."
• Do the disadvantaged women in BSP group get two types of cash transfers?
• How are we comparing the two cohorts when some of the study subjects in the control group enrolled in BISP are not eligible for cash transfers, while as everybody enrolled in BSP is, even though there are some specific conditions.

Response 2: Cash Transfer Asymmetry
We thank the reviewer for this important point and clarify the design further. Both cohorts are drawn from the same underlying population of Benazir Income Support Programme (BISP) beneficiaries, that is, women from the poorest households identified through the National Socio-Economic Registry. All women in both cohorts therefore receive the routine BISP unconditional quarterly cash transfer. The exposed cohort, by virtue of enrolment in the Benazir Nashonuma Programme (BNP), additionally receives the BNP package: specialised nutritious food, a BNP-specific conditional cash transfer provided quarterly (2,500 PKR (~USD 8.86) for pregnant women until childbirth. Post-delivery: 3,000 PKR (~USD 10.63) for a girl child and 2,500 PKR for a boy child) linked to compliance, antenatal care, and behaviour change communication. Specifically, women in the exposed cohort receive two cash streams (the BISP transfer and the BNP conditional transfer), whereas women in the unexposed cohort receive the BISP transfer only.

3. Is there any tool available to assess how the cash transfers are spent by the families because differences in the socio-economic status and other factors within the families can influence expenditure of this additional benefit.

Response 3: Expenditure Tracking
We thank the reviewer for this observation. Although the study does not employ a dedicated household expenditure ledger, the protocol captures several proximate indicators of how household resources, including the cash transfers, translate into food access and nutritional outcomes. At baseline and during follow-up we collect household food insecurity using the Food Insecurity Experience Scale (FIES) and maternal dietary quality using the Minimum Dietary Diversity for Women (MDD-W), alongside 24-hour dietary recall and detailed socio-demographic and socio-economic characteristics. Because socio-economic status and related household factors are recorded at baseline, they are adjusted for in the analysis and can be examined as potential effect modifiers of the relationship between programme exposure and nutritional outcomes. These measures allow us to assess whether additional income and supplementation are reflected in improved household food security and dietary diversity, even where individual-level expenditure is not itemized. A separate cost-benefit analysis will also allow us to assess cost-effectiveness of the program, and this intent has also been added to the protocol.

4. It appears the study is expected to be completed in June 2026. Was any Data Safety Monitoring Board (DSMP) meeting held during the study period? If yes, was there any concern raised by the board?

Response 4: DSMB
As the efficacy and effectiveness of the commodity is well established and the study aims to assess the impact of BNP on nutritional outcomes at the population level. Ethical oversight is maintained through annual progress reviews with AKU ERC
“The study is subject to annual continuing review and progress reporting to the Aga Khan University Ethics Review Committee (ERC No: 2021-6412-18742) and to oversight by the Pakistan National Bioethics Committee (NBC Reference No: 4-87/NBC-761/22/1654), with any adverse events reported in accordance with ERC requirements. No safety concerns have been raised through these mechanisms to date. We have clarified the rationale for this monitoring approach in the Ethical Considerations section.

VERSION 2 – REVIEW

**REVIEWER NAME**	Patwari, Ashok
**REVIEWER AFFILIATION**	Lady Hardinge Medical College, Pediatrics
**REVIEWER CONFLICT OF INTEREST**	No Conflict of Interest
**AI DISCLOSURE**	No, I have not used AI
**DATE REVIEW RETURNED**	14-Jul-2026

**GENERAL COMMENTS**	Thanks for clarifying some of the issues that I considered important for conducting this important study.
